# Assessment of Vascular Event Prevention and Cognitive Function Among Older Adults With Preexisting Vascular Disease or Diabetes: A Secondary Analysis of 3 Randomized Clinical Trials

**DOI:** 10.1001/jamanetworkopen.2019.0223

**Published:** 2019-03-01

**Authors:** Alison Offer, Matthew Arnold, Robert Clarke, Derrick Bennett, Louise Bowman, Richard Bulbulia, Richard Haynes, Jing Li, Jemma C. Hopewell, Martin Landray, Jane Armitage, Rory Collins, Sarah Parish

**Affiliations:** 1 Clinical Trial Service Unit and Epidemiological Studies Unit, Nuffield Department of Population Health, University of Oxford, Oxford, United Kingdom; 2 Medical Research Council Population Health Research Unit, Nuffield Department of Population Health, University of Oxford, Oxford, United Kingdom; 3 National Clinical Research Center of Cardiovascular Diseases, Fuwai Hospital, Beijing, China; 4 National Center for Cardiovascular Diseases, Chinese Academy of Medical Sciences and Peking Union Medical College, Beijing, China

## Abstract

**Importance:**

Acquisition of reliable randomized clinical trial evidence of the effects of cardiovascular interventions on cognitive decline is a priority.

**Objectives:**

To estimate the association of cognitive aging with the avoidance of vascular events in cardiovascular intervention trials and understand whether reports of nonsignificant results exclude worthwhile benefit.

**Design, Setting, and Participants:**

This secondary analysis of 3 randomized clinical trials in participants with preexisting occlusive vascular disease or diabetes included survivors to final in-trial follow-up in the Heart Protection Study (HPS), Study of the Effectiveness of Additional Reductions in Cholesterol and Homocysteine (SEARCH), and Treatment of HDL (High-Density Lipoprotein) to Reduce the Incidence of Vascular Events (HPS2-THRIVE) trials of lipid modification for prevention of cardiovascular events. Data were collected from February 1994 through January 2013 and analyzed from January 2015 through December 2018.

**Exposures:**

Incident vascular events and diabetes and statin therapy.

**Main Outcomes and Measures:**

Cognitive function was assessed at the end of a mean (SD) of 4.9 (1.5) years of follow-up using a 14-item verbal test. Associations of the incidence of vascular events and new-onset diabetes during the trials, with cognitive function at final in-trial follow-up were estimated and expressed as years of cognitive aging (using the association of the score with age >60 years). The benefit on cognitive aging mediated through the effects of lowering low-density lipoprotein cholesterol levels on events was estimated by applying these findings to nonfatal event differences observed with statin therapy in the HPS trial.

**Results:**

Among 45 029 participants undergoing cognitive assessment, mean (SD) age was 67.9 (8.0) years; 80.7% were men. Incident stroke (n = 1197) was associated with 7.1 (95% CI, 5.7-8.5) years of cognitive aging; incident transient ischemic attack, myocardial infarction, heart failure, and new-onset diabetes were associated with 1 to 2 years of cognitive aging. In HPS, randomization to statin therapy for 5 years resulted in 2.0% of survivors avoiding a nonfatal stroke or transient ischemic attack and 2.4% avoiding a nonfatal cardiac event, which yielded an expected reduction in cognitive aging of 0.15 (95% CI, 0.11-0.19) years. With 15 926 participants undergoing cognitive assessment, HPS had 80% power to detect a 1-year (ie, 20% during the 5 years) difference in cognitive aging.

**Conclusions and Relevance:**

The expected cognitive benefits of the effects of preventive therapies on cardiovascular events during even the largest randomized clinical trials may have been too small to be detectable. Hence, nonsignificant findings may not provide good evidence of a lack of worthwhile benefit on cognitive function with prolonged use of such therapies.

**Trial Registration:**

isrctn.com and ClinicalTrials.gov Identifiers: ISRCTN48489393, ISRCTN74348595, and NCT00461630

## Key Points

**Question:**

Do null results on cognitive function in cardiovascular trials exclude worthwhile benefit?

**Findings:**

In this secondary analysis of 3 randomized clinical trials including 45 029 participants undergoing cognitive assessment, the prevention of nonfatal cardiovascular events in 4.5% of survivors in the Heart Protection Study, by randomization to statin, yielded an estimated cognitive function difference equivalent to avoiding 0.15 years of aging. By contrast, the trial was powered to detect a difference in cognitive aging of at least 1 year.

**Meaning:**

Nonsignificant findings, even from large trials, should not be taken as good evidence of a lack of worthwhile benefit on cognitive function of prolonged use of cardioprotective therapies.

## Teaser

This secondary analysis of 3 randomized clinical trials estimates the effect on cognitive aging of the avoidance of vascular events and evaluates whether reports of nonsignificant results exclude worthwhile benefit among patients with vascular disease.

## Introduction

Dementia and cognitive impairment present major health care and social burdens that are increasing globally with increasing lifespan.^1^ Autopsy and neuroimaging studies show that many dementia cases involve vascular pathologic changes in the brain (such as infarcts, white matter lesions, and cerebral microbleeds).^2^ In numerous studies, higher levels of vascular risk factors in midlife and cardiovascular disease and diabetes are associated with future risk of dementia and cognitive decline.^3,4,5^ One important way that vascular risk factors (such as high blood pressure and high cholesterol levels) may cause cognitive impairment is by causing cerebrovascular events (such as stroke and transient ischemia).^3^ Conversely, one important way that interventions that reduce vascular risk factors may prevent dementia is by preventing cerebrovascular and other vascular events.

However, randomized clinical trials^6,7^ to prevent cardiovascular disease with therapies to lower blood pressure or low-density lipoprotein (LDL) cholesterol levels or antiplatelet therapies and that included cognitive assessment have failed to demonstrate benefits for cognition compellingly (eAppendix 1 and eTable 1 in the Supplement). Despite definitive evidence that such therapies prevent nonfatal vascular events, the absolute differences in the percentage of patients who have such events during a trial (typically 1%-5% of participants [eTable 1 in the Supplement]) may have been insufficient for plausible benefits on cognition to have been detected. An adequate assessment of the statistical power of the previous trials to detect the likely effects of cardiovascular disease prevention on cognition has not been presented. Specific therapies may also have effects on cognitive function through other mechanisms, but the focus of this report is solely on effects attributable to prevention of vascular events.

Participants recruited into the Heart Protection Study (HPS),^8^ Study of the Effectiveness of Additional Reductions in Cholesterol and Homocysteine (SEARCH),^9^ and Treatment of HDL to Reduce the Incidence of Vascular Events (HPS2-THRIVE)^10^ randomized clinical trials of therapies to modify lipid levels and who survived to the end of the in-trial follow-up represent a well-characterized population of 52 000 individuals in whom 9000 nonfatal cardiovascular or revascularization incident events occurred during a mean (SD) follow-up of 4.9 (1.5) years.^11,12,13,14^ The interventions in the SEARCH and HPS2-THRIVE studies did not result in statistically significant reductions in their primary vascular outcomes, but statin therapy in the HPS study resulted in a highly significant 24% proportional reduction in the incidence of major vascular events. The present report determines the associations between different types of vascular events that occurred during these 3 trials and cognitive function assessed at the end of follow-up. We use this association to estimate the effect on cognitive function expected from the reductions in vascular events (and increase in diabetes^15^) produced by statin therapy in HPS and compare that expected effect with the differences in cognitive function that the study was adequately powered to detect.

## Methods

### Study Design

The present analyses include the 51 974 participants in the HPS, SEARCH, and HPS2-THRIVE randomized clinical trials who survived until the final in-trial follow-up at the end of the scheduled treatment period. All 3 studies were used in the first stage of analysis to determine the association between incident events occurring during the trials and cognitive function assessed at the end of follow-up. However, only HPS resulted in a statistically significant reduction in the primary efficacy vascular outcome associated with statin therapy. Therefore, only HPS was suitable for the second stage of analysis to estimate the effect on cognitive function resulting from the vascular event reductions (and increase in diabetes^15^) produced by the intervention. These studies recruited participants with preexisting occlusive vascular disease or diabetes from the United Kingdom, as well as from Scandinavia and China in HPS2-THRIVE (Table 1 and eFigures 1 and 2 in the Supplement). Data for the trials were collected from February 1994 through January 2013. Approval was obtained from the ethics committees of the participating institutions for each of the studies, and all participants gave written informed consent, including for future analyses for medical research.

**Table 1.** Baseline Characteristics and Nonfatal Incidents in Trial Events in the 45 029 Participants With Cognitive Function Assessed at the Final Follow-up Visit

Characteristic	Randomized Clinical Trial				
	HPS (n = 20 536)	SEARCH (n = 12 064)	HPS2-THRIVE		All (n = 58 273)
			European Cohort (n = 14 741)	Chinese Cohort (n = 10 932)	
No. of randomized participants who survived to end of trial	17 701	10 130	14 046	10 097	51 974
Cognitive assessment at final follow-up, No. (%)	15 926 (90.0)	8879 (87.7)	12 310 (87.6)	7914 (78.4)	45 029 (86.6)
Characteristics of participants with cognitive assessment at final follow-up					
Age at entry, mean (SD), y	63.4 (8.4)	63.3 (8.6)	65.1 (7.1)	61.8 (7.2)	63.6 (8.0)
Age group age at entry, No. (%)					
<60 y	5165 (32.4)	3065 (34.5)	2816 (22.9)	3403 (43.0)	14 449 (32.1)
60-69 y	6741 (42.3)	3718 (41.9)	5952 (48.4)	3121 (39.4)	19 532 (43.4)
≥70 y	4020 (25.2)	2096 (23.6)	3542 (28.8)	1390 (17.6)	11 048 (24.5)
Age at cognitive assessment, mean (SD), y	68.2 (8.4)	69.8 (8.6)	68.0 (7.1)	65.2 (7.3)	67.9 (8.0)
Baseline characteristics					
Female, No. (%)	4120 (25.9)	1463 (16.5)	1739 (14.1)	1365 (17.2)	8687 (19.3)
Townsend deprivation index, mean (SD)^a^	−0.48 (3.18)	−0.94 (2.97)	NA	NA	−0.65 (3.11)
Systolic blood pressure, mean (SD), mm Hg	144 (23)	137 (21)	144 (20)	141 (22)	142 (22)
Current smoker, No. (%)	2065 (13.0)	975 (11.0)	1674 (13.6)	1873 (23.7)	6587 (14.6)
Current alcohol use, No. (%)	9606 (60.3)	5648 (63.6)	7803 (63.4)	1157 (14.6)	24 214 (53.8)
Prior disease at entry, No. (%)					
MI	6457 (40.5)	8879 (100)	8789 (71.4)	5405 (68.3)	29 530 (65.6)
Other CHD and no MI	8826 (55.4)	0	1102 (9.0)	1063 (13.4)	10 991 (24.4)
Peripheral vascular disease	4896 (30.7)	184 (2.1)	2111 (17.1)	340 (4.3)	7531 (16.7)
Cerebrovascular disease	2372 (14.9)	520 (5.9)	2911 (23.6)	2720 (34.4)	8523 (18.9)
Diabetes at entry	4505 (28.3)	815 (9.2)	2647 (21.5)	3024 (38.2)	10 991 (24.4)
LDL cholesterol level, mean (SD), mg/dL^b^	76.1 (23.9)	96.5 (23.2)	67.2 (16.6)	58.7 (15.8)	74.5 (24.3)
Nonfatal in-trial events, No. (%)					
Disabling stroke	203 (1.3)	22 (0.2)	24 (0.2)	37 (0.5)	286 (0.6)
Mild stroke (not disabling)	329 (2.1)	217 (2.4)	150 (1.2)	215 (2.7)	911 (2.0)
TIA	449 (2.8)	209 (2.4)	145 (1.2)	69 (0.9)	872 (1.9)
MI	662 (4.2)	565 (6.4)	369 (3.0)	224 (2.8)	1820 (4.0)
Coronary revascularization	1027 (6.4)	930 (10.5)	573 (4.6)	425 (5.4)	2955 (6.6)
Noncoronary revascularization	693 (4.4)	217 (2.4)	338 (2.7)	38 (0.5)	1286 (2.8)
Heart failure	352 (2.2)	208 (2.3)	136 (1.1)	263 (3.3)	959 (2.1)
Onset of diabetes^c^	545 (4.8)	1019 (12.6)	588 (6.1)	433 (8.8)	2585 (7.6)
TICS-m score at final follow-up, mean (SD)^d^	24.1 (4.2)	24.3 (4.1)	25.4 (3.9)	24.1 (4.2)	24.5 (4.2)
Verbal fluency score at final follow-up, mean (SD)^e^	21.5 (7.3)	22.5 (7.4)	23.3 (6.9)	19.4 (6.3)	21.8 (7.2)

Abbreviations: CHD, coronary heart disease; HPS, Heart Protection Study; HPS2-THRIVE, Treatment of HDL (High-Density Lipoprotein) to Reduce the Incidence of Vascular Events; LDL, low-density lipoprotein; MI, myocardial infarction; NA, not applicable; SEARCH, Study of the Effectiveness of Additional Reductions in Cholesterol and Homocysteine; TIA, transient ischemic attack; TICS-m, Modified Telephone Interview for Cognitive Status.

SI conversion factor: To convert cholesterol to millimoles per liter, multiply by 0.0259.

^a^ Only available in HPS and SEARCH. Ranges from −6.25 to 10.26, with higher scores indicating greater degree of deprivation.

^b^ At randomization in HPS (during simvastatin treatment, 40 mg/d) and SEARCH (during simvastatin treatment, 20 mg/d) and at the baseline visit in HPS2-THRIVE (during simvastatin treatment, 40 mg/d, with or without ezetimibe).

^c^ The denominator for the percentages excludes those with diabetes at entry.

^d^ Scores range from 0 to 39, with higher scores indicating greater cognitive ability.

^e^ Scores range from 0 to 72, with higher scores indicating greater verbal fluency.

The present analyses were conducted from January 2015 through December 2018. This study followed the Strengthening the Reporting of Observational Studies in Epidemiology (STROBE) reporting guidelines for observational studies. Baseline data recorded before randomization in each study included age, sex, smoking, alcohol use, prior disease, current medication use, height, weight, systolic and diastolic blood pressure, and measurements of blood lipid, lipoprotein, and creatinine levels.

### Follow-up

At regular follow-up visits until a participant’s scheduled final visit, information was sought from the participants about the occurrence of any serious adverse event. Further information was sought from additional sources, and more than 99% of participants had complete follow-up according to the trial procedures. Incident nonfatal events used in the analyses include stroke (subdivided by disability [eAppendix 2 in the Supplement]), transient ischemic attack (TIA), myocardial infarction (MI), heart failure, coronary and noncoronary revascularization procedures, and new-onset diabetes (see eAppendix 2 in the Supplement for further details).

### Cognitive Assessment

Cognitive function was assessed at the final follow-up visits in 45 029 surviving participants using the 13-item Modified Telephone Interview for Cognitive Status (TICS-m) with an additional verbal fluency test (translated into the local language for HPS2-THRIVE participants in Scandinavia and China).^14^ The TICS-m covers the component domains of orientation, memory (registration, recent memory, and delayed recall), attention/calculation, and language (semantic memory, comprehension, and repetition).

### Statistical Analysis

We analyzed HPS, SEARCH, and the European and Chinese arms of HPS2-THRIVE as 4 separate study cohorts, because the association of baseline risk factors with cognitive function might vary considerably between these regions. Global and domain-specific TICS-m scores and the verbal fluency scores in each study cohort were converted to *z* scores by subtracting the mean and dividing by the SD of the score within the cohort; global cognitive scores were formed from the TICS-m *z* score (with weight 4, because it covers 4 domains) plus the verbal fluency *z* score and similarly converted to a *z* score.

Analyses of the association of cognitive scores with age at test used linear regression, with adjustment for sex and prior disease (as indicated in eTable 2 in the Supplement). Baseline factors associated with cognitive function (at study end) were identified to allow adjustment for them in lieu of being able to allow for baseline cognitive function (which was not measured). These factors were identified separately in each of the 4 study cohorts by stepwise linear regression, with age at test (as single years), sex, and prior disease forced into the model and with the cutoff for selection/removal of the other (approximately 50) variables being 2-sided *P* = .001 (approximately .05 divided by the number of variables available for selection [eTable 2 in the Supplement]).

Associations of incident vascular events occurring during each study with the final visit cognitive function *z* scores were assessed by linear regression with the inclusion of randomized treatment assignment, duration of follow-up, baseline factors for cognitive function in the study cohort, and binary indicators for any incidence of each of the events of interest during the study. Results from the 4 study cohorts were combined in an inverse variance–weighted fixed-effects meta-analysis. Jackknife resampling was implemented to correct for bias and estimate SEs^16^; because the cognitive *z* scores were approximately normally distributed, *P* values were derived from jackknife estimates assuming normality. The inverse associations of cognitive *z* scores per year of age greater than 60 appeared to be approximately linear, and the slope in all cohorts combined was used to express differences in *z* scores as years of cognitive aging (eFigure 2, eFigure 3, and eAppendix 3 in the Supplement provides further rationale). Sensitivity analyses using alternative strengths of slopes were also conducted.

In HPS, the study mean reduction of 37 mg/dL (to convert to millimoles per liter, multiply by 0.0259) in LDL cholesterol level between participants randomized to simvastatin, 40 mg/d, vs placebo resulted in a highly significant 24% proportional reduction in the incidence of major vascular events (ie, MIs or coronary deaths, strokes, or revascularization procedures) during a mean scheduled treatment duration of 5 years.^11^ Large-scale meta-analyses of randomized clinical trials confirm that statin therapy prevents major vascular events and heart failure, but also indicate that statins increase the incidence of new-onset diabetes.^11,17,18^ Therefore, the estimated years of cognitive aging associated with each of these types of event were applied to the observed differences in the incidence of events between all statin- and placebo-assigned survivors in HPS to yield estimates of the expected cognitive aging associated with differences in event rates. Sensitivity analyses restricted to survivors with cognitive assessments were conducted to assess the effect of response bias. Expected differences in cognitive aging were compared with the observed differences (adjusted for baseline risk factors among survivors in the simvastatin and placebo arms [eAppendix 3 in the Supplement]). The magnitude of the effect on cognitive aging detectable with 80% power at *P* < .05 in HPS and the sample sizes needed to detect the expected effects were estimated using standard power calculation methods using R.

## Results

Cognitive assessment at final follow-up visits was available in 45 029 participants (mean [SD] age, 67.9 [8.0] years; 80.7% men and 19.3% women) across the 4 cohorts (86.6% of participants surviving to the end of the studies) (Table 1). The baseline prevalence of different types of vascular disease and risk factor levels varied across the studies (reflecting differences in study eligibility criteria and improvements in cardiovascular prevention over time), resulting in differing frequencies of incident events during the studies (Table 1). During a mean (SD) of 4.9 (1.5) years of follow-up, 1820 participants had at least 1 MI, 1197 had strokes, 872 had TIAs, 2585 developed diabetes, and 4112 had revascularization procedures. The 13.4% of surviving participants without cognitive assessments were slightly older than other survivors (mean [SD] age, 64.8 [8.9] vs 63.6 [7.9] years) and had somewhat higher event rates, especially stroke (8.4% vs 2.7%, compared with 3.4% overall [eTable 3 in the Supplement]).

### Baseline Factors Associated With Cognitive Function at Final Visit

**Figure 1.**
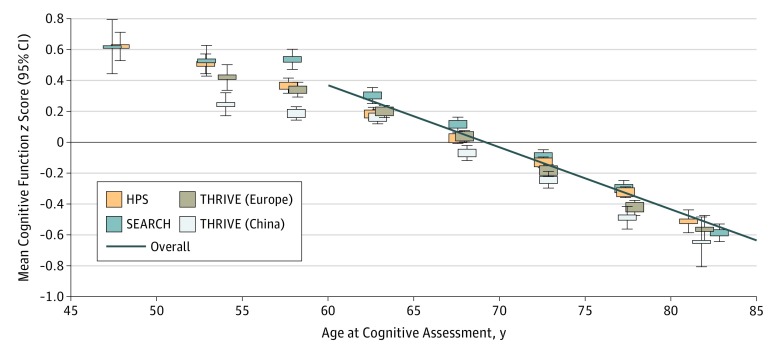
Mean Cognitive Function *z* Score by Age and Study Cohort Analyses are adjusted for age, sex, and baseline disease. In participants older than 60 years, the overall mean cognitive function was 4.0% (SE, 0.1%) of an SD lower per year of age, as shown by the diagonal solid line. The corresponding percentages in the separate cohorts were 3.6% (SE, 0.2%) SD in the Heart Protection Study (HPS), 4.4% (SE, 0.2%) SD in the Study of the Effectiveness of Additional Reductions in Cholesterol and Homocysteine (SEARCH), 4.2% (SE, 0.2%) SD in the European cohort of the Treatment of HDL (High-Density Lipoprotein) to Reduce the Incidence of Vascular Events (HPS2-THRIVE), and 4.0% (SE, 0.1%) SD in the Chinese cohort of HPS2-THRIVE. Whiskers represent 95% CIs.

The global cognitive function *z* scores were normally distributed and, at older than 60 years, had inverse linear associations with age within each of the 4 study cohorts, and were 0.04 (SE, 0.0008) lower per year (ie, 4% of an SD per year overall [Figure 1]). In addition to age, shorter height, sex (male in Europe and female in China), and baseline cerebrovascular disease, diabetes, or higher hemoglobin A_1c_ levels were strong independent risk factors for lower cognitive function in all cohorts (eTable 3 and eTable 4 in the Supplement, which gives the step number, effect size, and percentage of the sum of squares explained for each factor as indications of the relevance of different factors). Further strong independent risk factors for lower cognitive function were Townsend deprivation index (derived from postcode in the United Kingdom only), lower levels of alcohol consumption, higher homocysteine levels in SEARCH (the only cohort in which it was measured), and not taking aspirin (eTable 4 in the Supplement).

### Cognitive Function Differences Associated With Incident Events

**Figure 2.**
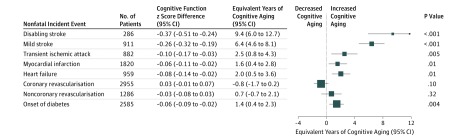
Cognitive Aging Associated With Nonfatal Incident Events Among 45 029 participants in the Heart Protection Study (HPS), Study of the Effectiveness of Additional Reductions in Cholesterol and Homocysteine (SEARCH), and Treatment of HDL (High-Density Lipoprotein) to Reduce the Incidence of Vascular Events trials, analyses were adjusted for age, sex, baseline disease, randomized treatment allocation, duration in study, and the baseline risk factors associated with cognitive function in each trial.

The global cognitive function *z* score differences and the corresponding equivalent years of cognitive aging associated with the incidence of different events are shown in Figure 2. Occurrence of at least 1 mild stroke was associated with a *z* score difference of −0.26 (95% CI, −0.32 to −0.19), corresponding to 6.4 (95% CI, 4.6-8.1; *P* < .001) years of cognitive aging, whereas the quarter of incident strokes classified as disabling were associated with 9.4 (95% CI, 6.0-12.7; *P* < .001) years of greater cognitive aging, yielding an overall effect for strokes of any severity of 7.1 (95% CI, 5.7-8.5) years. Incident TIA was associated with 2.5 (95% CI, 0.8-4.3; *P* = .005) years of cognitive aging; MI, 1.6 (95% CI, 0.4-2.8; *P* = .01) years; heart failure, 2.0 (95% CI, 0.5-3.6; *P* = .01) years; and new-onset diabetes, 1.4 (95% CI, 0.4-2.3; *P* = .004) years. Revascularization procedures were not associated with significant effects.

In subgroup analyses for cerebrovascular events, the pattern of cognitive aging associated with events was similar for recent (within 2 years before cognitive testing) and earlier events (eFigure 4 in the Supplement), and in participants younger and older than 70 years (with approximately half the events in each age bracket). Too few recurrent strokes were recorded to assess the additional association with multiple strokes (eTable 3 in the Supplement).

### Association of Event Prevention With Cognitive Function

The absolute percentage of survivors in HPS avoiding nonfatal events through assignment to statin therapy was 1.97% for cerebrovascular events (0.68% disabling stroke, 0.64% mild stroke, and 0.74% TIA) and 2.40% for all cardiac events (and 4.53% for all cardiovascular events). Table 2 gives the effects for these and other events.

**Table 2.** Estimated Benefit for Cognitive Function of the Effects of Statin Therapy on Events in the Heart Protection Study^a^

Nonfatal Events	Patients With Event, No. (%)		Avoidance of Events With Statin, %^b^	Higher Cognitive Function *z* Score (SE)		Reduction in Years of Cognitive Aging (SE)	
	Randomized to Simvastatin	Randomized to Placebo		Per Event Avoided	Per Person	Per Event Avoided	Per Person^c^
**All Participants Surviving to the End of the Trial (n = 17 701)**^d^							
Disabling stroke	122 (1.4)	176 (2.0)	0.68	0.374 (0.068)	0.0025 (0.0005)	9.4 (1.7)	0.063 (0.012)
Mild stroke	178 (2.0)	229 (2.6)	0.64	0.255 (0.035)	0.0016 (0.0002)	6.4 (0.9)	0.041 (0.006)
TIA	226 (2.5)	284 (3.2)	0.74	0.102 (0.036)	0.0008 (0.0003)	2.6 (0.9)	0.019 (0.007)
All cerebrovascular events^e^	487 (5.4)	634 (7.2)	1.97	0.731 (0.085)	0.0049 (0.0006)	18.3 (2.1)	0.123 (0.014)
MI	292 (3.3)	464 (5.3)	2.22	0.063 (0.024)	0.0014 (0.0005)	1.6 (0.6)	0.035 (0.013)
Heart failure	206 (2.3)	219 (2.5)	0.22	0.081 (0.032)	0.0002 (0.0001)	2.0 (0.8)	0.004 (0.002)
All cardiac events^e^	466 (5.2)	642 (7.3)	2.40	0.144 (0.040)	0.0016 (0.0005)	3.6 (1.0)	0.039 (0.013)
New-onset diabetes	314 (4.9)	272 (4.4)	−0.60	0.055 (0.019)	−0.0003 (0.0001)	1.4 (0.5)	−0.008 (0.003)
Net effect estimated from events avoided	NA	NA	NA	0.930 (0.095)	0.0062 (0.0008)	23.3 (2.4)	0.154 (0.020)
**Participants With Cognitive Assessment Surviving to Final Follow-up (n = 15 926)**^f^							
Disabling stroke	84 (1.0)	119 (1.5)	0.50	0.374 (0.068)	0.0019 (0.0003)	9.4 (1.7)	0.047 (0.009)
Mild stroke	142 (1.8)	187 (2.4)	0.66	0.255 (0.035)	0.0017 (0.0002)	6.4 (0.9)	0.042 (0.006)
TIA	201 (2.5)	248 (3.2)	0.73	0.102 (0.036)	0.0007 (0.0003)	2.6 (0.9)	0.019 (0.007)
All cerebrovascular events^e^	395 (4.9)	509 (6.5)	1.79	0.731 (0.085)	0.0043 (0.0005)	18.3 (2.1)	0.107 (0.012)
MI	251 (3.1)	411 (5.2)	2.36	0.063 (0.024)	0.0015 (0.0006)	1.6 (0.6)	0.037 (0.014)
Heart failure	172 (2.1)	180 (2.3)	0.21	0.081 (0.032)	0.0002 (0.0001)	2.0 (0.8)	0.004 (0.002)
All cardiac events^e^	395 (4.9)	555 (7.1)	2.52	0.144 (0.040)	0.0017 (0.0006)	3.6 (1.0)	0.041 (0.014)
New-onset diabetes	293 (5.1)	252 (4.5)	−0.60	0.055 (0.019)	−0.0003 (0.0001)	1.4 (0.5)	−0.008 (0.003)
Net effect estimated from events avoided	NA	NA	NA	0.930 (0.095)	0.0056 (0.0008)	23.3 (2.4)	0.141 (0.019)

Abbreviations: MI, myocardial infarction; NA, not applicable; TIA, transient ischemic attack.

^a^ Study mean reduction in low-density lipoprotein cholesterol level was 37 mg/dL (to convert to millimoles per liter, multiply by 0.0259).

^b^ Estimated with adjustment for age and major vascular event risk score (as described in eAppendix 3 in the Supplement). Although the study treatment arms were well balanced for age and risk factors at randomization, the excess probability of surviving in the simvastatin arm compared with the placebo arm was greater in higher-risk participants, resulting in an imbalance in baseline risk factors between the allocated treatment arms among survivors to the end of the study, necessitating adjustment.

^c^ For comparison, the observed reduction in cognitive aging with randomization to simvastatin vs. placebo was 0.35 (95%CI, −0.37 to 1.06) years, estimated with adjustment for duration in trial, baseline risk factors for cognitive function and major vascular event risk score (eAppendix 3 in the Supplement).

^d^ Includes 8942 in the simvastatin group and 8759 in the placebo group.

^e^ Numbers of patients with events in the summary categories are less than the sums of the numbers for the component events as some patients had more than one type of event; but the cognitive function effects and reductions in cognitive aging shown for the summary categories are the sums of those for the contributing components.

^f^ Includes 8088 in the simvastatin group and 7838 in the placebo group.

Based on the associations in Figure 2 for events affected by statin therapy, these observed event differences would be expected to result in a 0.0062 (SE, 0.0008) higher cognitive function *z* score or a reduction of 0.154 (SE, 0.020) years in cognitive aging among all survivors (ie, including those who did not complete cognitive testing [Table 2]). Among the participants with cognitive assessments (a subset that may be biased against inclusion of individuals with a disabling stroke or cognitive impairment), the expected reduction in cognitive aging was 0.141 (SE, 0.019) years (ie, about 10% smaller than the estimate among all survivors [Table 2]). The directly observed effect on cognitive function of randomization to statin therapy in HPS (which would reflect not only any effects mediated via event reductions but any other effects of a statin on cognition, and which can only be assessed among those completing cognitive assessment) was a difference of 0.35 (95% CI, −0.37 to 1.06; *P* = .35) years of cognitive aging (deriving from a difference of 0.014 [95% CI, −0.015 to 0.043] in the *z* score). This observed effect on cognitive aging had wide confidence limits encompassing both estimates of the expected effect (Table 2; eAppendix 3 and eTable 5 in the Supplement).

The assumed rate of cognitive decline with age affects the estimates of the expected and observed effects on cognitive aging to the same extent, but it does not affect their relative values. Sensitivity analyses assuming different rates of cognitive decline with age (3%-5% of an SD per year) resulted in reductions in expected cognitive aging varying from 0.19 to 0.11 years and observed differences varying from 0.46 to 0.28 years (eTable 6 in the Supplement).

## Discussion

These analyses of cognitive function measures in 45 029 individuals with vascular disease have allowed quantification of the association of cardiovascular events and diabetes with cognitive function during aging and estimation of the expected benefit on cognitive aging of a therapy that prevents such cardiovascular events. The expected benefit on cognitive function in HPS through the effects of statin treatment on such events was a reduction in cognitive aging of only about 0.15 years. An effect of this size is encompassed by, but small in comparison with, the 95% CI (−0.37 to 1.06 years) around the reduction of 0.35 years in cognitive aging observed with assignment to statin therapy, indicating that the HPS did not have sufficient statistical power to detect the expected benefit. Consequently, as with similar results for cardiovascular risk factor modification strategies in other trials^18,19,20^ (eTable 1 in the Supplement), HPS does not provide good evidence of a lack of beneficial effects of these strategies on cognitive function.

Any benefits on cognition are likely to derive from avoidance of irreversible adverse effects on the brain that would accumulate with duration of treatment. In clinical practice, therapy to lower LDL levels is intended to be taken for much longer than in randomized clinical trials. Moreover, the use of other measures that protect against cardiovascular events (such as cessation of tobacco use, blood pressure lowering, and antiplatelet therapy) can contribute to avoidance of vascular events. Benefit may be derived from avoidance not only of overt clinical events but also of subclinical events, such as silent strokes (which have been shown by brain imaging studies to be several times more frequent than symptomatic strokes), as well as TIAs that go unreported by patients.^21,22^ Because longitudinal studies suggest that preclinical pathologic changes related to dementia may begin decades before the onset of dementia, a long potential window for preventive intervention exists.^23^ Consequently, the combined effect on cognitive function of the avoidance of clinical and subclinical cardiovascular events with the use of multiple vascular protective interventions from midlife onward might well be many times bigger than that estimated to occur with lowering of LDL levels alone during a 5-year trial.

Most of the expected benefit of vascular event avoidance on cognitive aging in the present study derived from the avoidance of nonfatal cerebrovascular events in 2.0% of survivors, which contributed an expected 0.12 (SE, 0.01) years of reduction in cognitive aging, whereas avoidance of cardiac events in 2.4% of survivors contributed an expected 0.04 (SE, 0.01) years of reduction in cognitive aging (Table 2, subtotals for cerebrovascular and cardiac events, respectively). The cognitive aging found to be associated with MI, heart failure, and diabetes may in part be mediated through increased risk of subclinical cerebrovascular events. Incomplete adjustment for disease status at baseline might also contribute small differences in cognitive aging associated with event risk. In particular, a measure of glycemic status in patients without diabetes was available only in HPS2-THRIVE. Previous studies have found diabetes to be associated with approximately a 20% increase in the rate of cognitive aging, which is somewhat lower than the estimate in the present study.^5^ By either estimate, the expected adverse effect of statin on cognitive aging associated with new-onset diabetes is small in comparison with the expected benefit mediated through avoidance of cerebrovascular events. However, the uncertainty in the effect of the increase in diabetes onset highlights the need to be able to detect modest effects on cognitive function from the avoidance of vascular events, as well as the effects of any hazards, in randomized clinical trials of cardioprotective therapies given for only a limited duration.

The TICS-m plus verbal fluency tests are practical to administer to all participants in large cardiovascular prevention trials and are sufficient to detect any large effects on cognition. For example, in HPS, the combined test had 80% power (at 2-sided *P* < .05) to detect a 1-year difference in cognitive aging (ie, a 20% proportional difference during the 5 years of the study) between the treatment arms. Some cognitive domains (eg, reaction time and visuospatial processing) are not assessed by the TICS-m test, and more extensive test batteries tend to perform better. However, because they take longer to complete, they have tended to be used only in smaller studies or in subsets of larger studies, which reduces any advantage of the greater sensitivity of these tests for the assessment of effects on cognitive function.^24^ For example, the FOURIER (Further Cardiovascular Outcomes Research with PCSK9 in Subjects With Elevated Risk) randomized clinical trial of evolocumab assessed cognition with the Cambridge Neuropsychological Test Automated Battery^25^ at baseline and 1 or more times during follow-up in a subset of 1204 patients followed up for 19 months.^26^ The SE of the difference between treatment arms in the change in the primary cognition outcome *z* score suggests that the study only had 80% power (at 2-sided *P* < .05) to detect approximately a 3-year (200%) difference in cognitive aging (eAppendix 3 in the Supplement).

Although more extensive cognitive testing might not previously have been feasible in large clinical trials (and may have adversely affected their ability to achieve the primary aims), electronic-device–based test batteries that can be self-administered at home and repeated readily on several occasions^27,28^ may now allow cognitive decline to be assessed with sufficient accuracy to detect effects of vascular event prevention in randomized clinical trials that are large enough and long enough. The SE in the FOURIER comparison is approximately 40% smaller than that expected from an end *z* score comparison, suggesting that if HPS had used such a test approach, then it might have had power to detect approximately a 12% proportional difference in cognitive aging (eAppendix 3 in the Supplement). This difference may be the level of benefit plausible in a trial with a primary focus on preventing cognitive decline, but it is about 4 times the proportional difference of about 3% (equivalent to 0.15 years of cognitive aging over 5 years) that is expected from the avoidance of cardiovascular events in the HPS trial. To detect the effect would, however, require 15 to 20 times more participant years of exposure than in HPS.

### Limitations

The study was limited by only having cognitive function measured at the study end and therefore could only use end of study cognitive function (rather than change in cognitive function) as an outcome. Furthermore, the TICS-m test used does not cover all cognitive domains. Against these limitations, the simpler testing allowed a much larger number of participants to undergo cognitive assessment than has been achieved in many randomized clinical trials.

## Conclusions

Occlusive vascular events are associated with reductions in cognitive function. However, the benefits on cognitive function likely to result from the effects of individual cardioprotective therapies on the occurrence of such events during the course of a typical trial of limited duration are likely to have been too small to be detected by the cognitive testing strategies previously considered feasible. Nevertheless, combined use of multiple vascular-protective interventions from midlife onward might prevent several years of cognitive aging. Novel strategies to assess decline in cognitive function more precisely that are feasible for use in large-scale randomized clinical trials may allow direct evidence about these benefits to emerge.

## Notes

Supplement. **eAppendix 1.** Evidence Before This Study **eAppendix 2.** Further Details of the Study Design **eAppendix 3.** Further Statistical Methods **eTable 1.** Randomized Clinical Trials of Therapy to Lower Blood Pressure or Lipid Levels, or Antiplatelet Therapy With Statistically Significant Effects on the Primary Cardiovascular End Point, Nonfatal Stroke or Nonfatal Myocardial Infarction, That Also Report on Cognition **eTable 2.** Baseline Factors Considered in Each Study **eTable 3.** Baseline Characteristics and Incident Nonfatal In-Trial Events in the 45 029 Participants With Cognitive Function Assessed at the Final Follow-up Visit and 6944 Participants Surviving to the End of the Trial But With No Cognitive Function Assessment **eTable 4.** Independent Baseline Risk Factors Associated With Cognitive Function in Each Trial From Stepwise Regression **eTable 5.** Cognitive Function *z* Score Differences and Cognitive Aging by Randomization to Simvastatin vs Placebo in the Heart Protection Study, Showing the Importance of Adjustment **eTable 6.** Sensitivity Analysis to Show the Effect of Assuming Different Rates of Declines in Cognitive Function With Age **eFigure 1.** Study Profiles of Cohorts Included in the First Stage of the Analysis **eFigure 2.** Study Profile by Randomization Arm of HPS, the Only Study Eligible for the Second Stage of the Analysis **eFigure 3.** Associations of the Components of the Global Cognitive Function Score With Age **eFigure 4.** Cognitive Aging Associated With the Incidence of Nonfatal Cerebrovascular Events by Time From Event to Assessment and by Age at Assessment **eReferences.**
