# Typographical Error in Title

**DOI:** 10.1001/jamanetworkopen.2019.2236

**Published:** 2019-03-15

In the Original Investigation titled “Assessment of Vascular Event Prevention and Cognitive Function Among Older Adults With Preexisting Vascular Disease or Diabetes: A Secondary Analysis of 3 Randomized Clinical Trials,”^1^ published March 1, 2019, a typographical error in the title was present on initial publication. This article has been corrected.^1^
