# Supplementary material for: Assessment of Vascular Event Prevention and Cognitive Function Among Older Adults With Preexisting Vascular Disease or Diabetes: A Secondary Analysis of 3 Randomized Clinical Trials
Source: JAMA Netw Open. 2019 Mar 1;2(3):e190223. doi: 10.1001/jamanetworkopen.2019.0223 (PMC6484650; doi:10.1001/jamanetworkopen.2019.0223)
Supplement: Supplement. — eAppendix 1. Evidence Before This Study eAppendix 2. Further Details of the Study Design eAppendix 3. Further Statistical Methods eTable 1. Randomized Clinical Trials of Therapy to Lower Blood Pressure or Lipid Levels, or Antiplatelet Therapy With Statistically Significant Effects on the Primary Cardiovascular End Point, Nonfatal Stroke or Nonfatal Myocardial Infarction, That Also Report on Cognition eTable 2. Baseline Factors Considered in Each Study eTable 3. Baseline Characteristics and Incident Nonfatal In-Trial Events in the 45 029 Participants With Cognitive Function Assessed at the Final Follow-up Visit and 6944 Participants Surviving to the End of the Trial But With No Cognitive Function Assessment eTable 4. Independent Baseline Risk Factors Associated With Cognitive Function in Each Trial From Stepwise Regression eTable 5. Cognitive Function z Score Differences and Cognitive Aging by Randomization to Simvastatin vs Placebo in the Heart Protection Study, Showing the Importance of Adjustment eTable 6. Sensitivity Analysis to Show the Effect of Assuming Different Rates of Declines in Cognitive Function With Age eFigure 1. Study Profiles of Cohorts Included in the First Stage of the Analysis eFigure 2. Study Profile by Randomization Arm of HPS, the Only Study Eligible for the Second Stage of the Analysis eFigure 3. Associations of the Components of the Global Cognitive Function Score With Age eFigure 4. Cognitive Aging Associated With the Incidence of Nonfatal Cerebrovascular Events by Time From Event to Assessment and by Age at Assessment eReferences. [file jamanetwopen-2-e190223-s001.pdf]

Supplementary Online Content

Offer A, Arnold M, Clarke R, et al; Heart Protection Study (HPS), Study of the Effectiveness of Additional Reductions in Cholesterol and Homocysteine (SEARCH), and Treatment of HDL (High-Density Lipoprotein) to Reduce the Incidence of Vascular Events (HPS2-THRIVE) Collaborative Groups. Assessment of vascular event prevention and cognitive function among older adults with preexisting vascular disease or diabetes: a secondary analysis of 3 randomized clinical trials. *JAMA Netw Open*. 2019;2(3):e190223. doi:10.1001/jamanetworkopen.2019.0223

eAppendix 1. Evidence Before This Study

eAppendix 2. Further Details of the Study Design

eAppendix 3. Further Statistical Methods

eTable 1. Randomized Clinical Trials of Therapy to Lower Blood Pressure or Lipid Levels, or Antiplatelet Therapy With Statistically Significant Effects on the Primary Cardiovascular End Point, Nonfatal Stroke or Nonfatal Myocardial Infarction, That Also Report on Cognition

eTable 2. Baseline Factors Considered in Each Study

eTable 3. Baseline Characteristics and Incident Nonfatal In-Trial Events in the 45 029 Participants With Cognitive Function Assessed at the Final Follow-up Visit and 6944 Participants Surviving to the End of the Trial But With No Cognitive Function Assessment

eTable 4. Independent Baseline Risk Factors Associated With Cognitive Function in Each Trial From Stepwise Regression

eTable 5. Cognitive Function z Score Differences and Cognitive Aging by Randomization to Simvastatin vs Placebo in the Heart Protection Study, Showing the Importance of Adjustment

eTable 6. Sensitivity Analysis to Show the Effect of Assuming Different Rates of Declines in Cognitive Function With Age

eFigure 1. Study Profiles of Cohorts Included in the First Stage of the Analysis

eFigure 2. Study Profile by Randomization Arm of HPS, the Only Study Eligible for the Second Stage of the Analysis

eFigure 3. Associations of the Components of the Global Cognitive Function Score With Age

eFigure 4. Cognitive Aging Associated With the Incidence of Nonfatal Cerebrovascular Events by Time From Event to Assessment and by Age at Assessment

eReferences.

© 2019 Offer A et al. *JAMA Network Open.*

This supplementary material has been provided by the authors to give readers additional information about their work.

© 2019 Offer A et al. *JAMA Network Open.*

Supplementary Methods

eAppendix 1: Evidence Before This Study

Evidence from randomized trials of the effects of cardiovascular event prevention with blood-pressure-lowering, lipid-lowering, or antiplatelet therapy on cognitive function or dementia was systematically reviewed. Review was restricted to cardiovascular outcome trials that successfully prevented cardiovascular events, defined as having a statistically significant effect on their primary cardiovascular outcome or on non-fatal stroke or non-fatal myocardial infarction (MI) presented in the main report of the trial, and published before 30th August 2017. Trials specifically in demented populations were excluded. For lipid-lowering and blood-pressure lowering, trials were identified from recent systematic reviews[1-5] of the effects of such therapies on cognitive function, together with expert review by the authors to add any more recent such trials (which would be well known). For antiplatelet therapy, we undertook a search restricting to randomized trials, using the terms 'aspirin' or 'antiplatelet*' in titles or abstracts combined with cogniti*, dement* or memory in titles or abstracts or the MeSH term cognition. Relevant reports were searched for mention of additional studies before restricting to cardiovascular outcome studies as for the other therapies. For blood pressure-lowering therapy, from 11 trials in three reviews, seven met the outcome criteria;[6-20] for lipid lowering therapy from 15 trials in two reviews plus five trials from expert review, five met the outcome criteria;[21-26] for antiplatelet therapy, from 43 initial hits, two trials met the outcome criteria.[27-30]

Overall, most trials failed to show a benefit of therapy on cognition (*eTable 1*). The two largest trials of cognitive function (about 25 000 survivors in the REVEAL trial of anacetrapib and 15 926 survivors in the Heart Protection Study of statin therapy) failed to show such benefit, but reductions in non-fatal events were only 0·7% for MI and <0·1% for stroke in REVEAL, and 2·2 % for MI and 1·1 % for stroke in HPS. The greatest reductions in non-fatal events (4-5%) were observed in the blood pressure-lowering trials, some of which did observe a statistically significant effect on a cognitive measure. However, the evidence was inconclusive overall, as concluded in a recent review on the impact of hypertension on cognitive function.[31]

© 2019 Offer A et al. *JAMA Network Open.*

eAppendix 2: Further Details of the Study Design

Full details of the designs of the Heart Protection Study (HPS), SEARCH, and HPS2-THRIVE studies, including their protocols[32-34], have been reported previously.[21,22,34-37] Patients were eligible for the HPS randomized trial if they had a non-fasting blood total cholesterol concentrations of at least 135mg/dL (3.5 mmol/L) and a history of coronary disease, ischemic stroke, other occlusive disease of the non-coronary arteries, diabetes mellitus, or (if men aged ≥65 years) treated hypertension, and were aged between 40 and 80 at randomization. HPS randomized 20 536 participants in the UK to 40 mg simvastatin daily or placebo, and separately in a 2x2 factorial design to antioxidant vitamins or placebo. Patients were eligible for SEARCH if they had a history of myocardial infarction and were aged between 18 and 80 at randomization. SEARCH, randomized 12 064 participants in the UK to 80 versus 20 mg simvastatin daily, and separately in a 2x2 factorial design to folic acid plus vitamin B_{12} or placebo. Patients were eligible for HPS2-THRIVE if they had a history of MI, cerebrovascular disease, peripheral arterial disease, or diabetes mellitus with evidence of symptomatic coronary disease and were aged between 50 and 80 at randomization. In HPS2-THRIVE, 14 741 European and 10 932 Chinese participants were to receive 2 g niacin with 40 mg laropiprant daily versus placebo (on top of 40 mg simvastatin daily plus, when required, 10 mg ezetimibe) (*eFigure 1*). Baseline data recorded prior to randomization in each study included age, sex, smoking and alcohol use, history of prior disease, current medication use, height, weight, systolic and diastolic blood pressure and blood measurements of lipids, lipoproteins and creatinine.

Participants in all three trials were followed up for the incidence of events with follow-up visits every six months after the first year, and more frequently during the first year. Participants in the HPS, SEARCH and HPS2-THRIVE were followed up for a mean of 5·0, 6·7 and 3·9 years, respectively. Information was recorded at each visit about any serious adverse event. Where patients did not attend a follow-up visit they were followed up by telephone or through their general practitioners (GPs). Further information was sought from GPs or hospital records and events were coded by co-ordinating center clinicians blinded to the treatment allocation in the trial. Severity of stroke was classified as mild where there seemed to be no help needed with everyday activities. [21,22,34-37] For the analyses presented here, in HPS and SEARCH, onset of diabetes was defined as any hospitalisation for diabetes or taking oral hypoglycaemic mediation or insulin at any time during the trial (in SEARCH) or at the final visit (in HPS) in patients not diabetic at randomization. In HPS2-THRIVE,

© 2019 Offer A et al. *JAMA Network Open.*

diabetes at baseline was defined as: self-report or baseline plasma glucose ≥200 mg/dL if fasted <8 hours or ≥126 mg/dL if fasted ≥8 hours, or baseline HbA1c ≥48 mmol/mol, or use of hypoglycaemic medication at randomization. Self-reported diabetes and use of hypoglycaemic medications were recorded directly at each visit. Onset of diabetes was defined as self-report or new use of hypoglycaemic medication.

Approval was obtained from the ethics committees of the participating institutions for each of the studies, and all participants gave written informed consent.

eAppendix 3: Further Statistical Methods

Expression of z-score differences as years of cognitive aging

Cognitive measures typically decline steadily with age at least from about age 60 onwards.[38,39] At younger ages relationships are less consistent and may not reflect aging. In the present study, mean age at cognitive assessment was 64 years and 68% of participants were aged over 60 at cognitive assessment. Therefore the slope of the relationship between the cognitive measures in different participants and their age in those above age 60 (which was linear) was used to express z-scores differences as years of cognitive aging.

Comparisons between simvastatin versus placebo allocated survivors in HPS: The excess probability of surviving in the simvastatin arm compared to the placebo arm was greater in higher risk participants, resulting in an imbalance in baseline risk factors between the allocated treatment arms amongst survivors to the end of the study. Comparisons of incident events during the trial in survivors were therefore adjusted for age and baseline major vascular event risk (using a previously reported grouping[40]). Non-fatal events avoided per person with statin were estimated as (1/(odds ratio for the event in the statin versus placebo arm) -1) x proportion with event in the statin arm. In the main analyses in this report , the observed reduction in cognitive aging between the simvastatin and placebo allocated participants in HPS (*Table 2*) was adjusted for duration in trial, baseline predictors of cognitive function (for consistency with the analyses of the cognitive aging) and major vascular event risk (to allow for differences between the survivors in the two study arms). In supplementary analyses both unadjusted analyses (as in the original trial report) and adjusted analyses are presented. All analyses were conducted using SAS version 9.4.

© 2019 Offer A et al. *JAMA Network Open.*

Deductions from the Evolocumab trial

In the Further Cardiovascular Outcomes Research with PCSK9 in Subjects with Elevated Risk (FOURIER) report,[42] the treatment difference in the change in z-score for the primary end point had a standard error of about 0.036 (taken from the confidence interval in Figure 2 of the report) among the primary analysis population of 618 allocated placebo and 586 allocated Evolocmab. This is 38% smaller than the expected standard error from the treatment difference in an end z-score (which would be $\sqrt{[1/618+1/586]}=0.058$, assuming the z-score had standard deviation 1).

© 2019 Offer A et al. *JAMA Network Open.*

eTable 1. Randomized Clinical Trials of Therapy to Lower Blood Pressure or Lipid Levels, or Antiplatelet Therapy With Statistically Significant Effects on the Primary Cardiovascular End point, Nonfatal Stroke or Nonfatal Myocardial Infarction, That Also Report on Cognition

Randomized trial	Number in trial	Primary outcome	P value primary outcome/ non-fatal stroke / non-fatal MI	Number in cognitive study	Basis of cognitive assessment	Cognitive follow up (years)	Estimated % of active arm of cognitive study avoiding non-fatal stroke / MI[a]	Direction[b] of effect and p value
**Blood pressure lowering**								
PROGRESS[6,7]	6105	Stroke	<0.001 / <0.001 / 0.004	5888	MMSE	3.9	3.5 / 1.2	+0.01
					Dementia	3.9	3.5 / 1.2	NS
SHEP[8,9]	4736	Stroke	<0.001 / <0.001 / 0.03	4608	Short-CARE	5.0	2.5 / 0.5	+0.05
SCOPE[10,11]	4964	MVE	NS / 0.04 / NS	4835	MMSE	3.7	1.1 /-0.3	NS
SCOPE substudy[12]				228	CDR + 2 tests[c]	3.7	3.7 / 1.8	+0.04
SYST-EUR[13,14]	4695	Stroke	0.003 / 0.007 / NS	2418	Dementia	2.0	1.0 / 0.3	+0.05
					MMSE	2.0	1.0 / 0.3	NS
MRC[15,16]	4396	Stroke	0.04 / 0.03 / NS	2564	2 tests	4.5	1.0 / 0.2	NS
ACCORD[17,18]	4733	MVE	NS / 0.03 / NS	1349	MMSE + 3 tests	3.3	0.7 / 0.4	NS
HYVET[19,20]	3845	Stroke	0.009 / NS / NS	3336	MMSE, dementia	2.2	0.2 / 0.2	NS
**Lipid lowering**								
HPS[21]	20536	MVE	<0.001 / <0.001 / <0.001	15926	TICS-m + 1 test	5.3	1.1 / 2.2	NS
SEARCH[22]	12064	MVE	NS / NS / 0.02	8879	TICS-m + 1 test	7.1	0.4 / 1.0	NS
FOURIER/ EBBINGHAUS[23,24]	27564	MVE	<0.001 / <0.001[d] / <0.001[d]	1204	CANTAB	1.6	0.3 / 0.9	NS
PROSPER[25]	5804	MVE	0.014 / NS / NS	survivors	MMSE + 2 tests	3.2	0.1 / 1.0	NS
REVEAL[26]	30449	MCE	0.004 / NS / 0.007[d]	~25000	TICS-m	4.1	<0.1 / 0.7	NS
**Antiplatelet**								
MRC TPT[27,28]	5085	IHD	0.04[e] / NS / 0.004[e]	405	Global (5 tests)	5+	0.4 / 1.7	NS[e]
Womens Health Study[29,30]	39876	MVE	NS / 0.02 / NS	6377[f]	Global (TICS + 2 tests)	4.0	− / −	NS[g]

MI=myocardial infarction, MVE=major vascular event (cardiovascular death, non-fatal myocardial infarction, non-fatal stroke [in HPS, SEARCH and FOURIER it also includes revascularisation procedures, FOURIER also includes hospitilisation for angina]), MCE=major coronary event (coronary death, myocardial infarction, coronary revascularisation), IHD=ischemic heart disease, MMSE=mini mental state examination, CDR=cognitive drug research computerized assessment battery, TICS(-m)=(modified) telephone interview for cognitive status, CANTAB=Cambridge neurolophysological test automated battery, NS=not significant.

[a] Not directly reported, estimated from the absolute rate of non-fatal events in each arm and / or the overall risk reduction as available, except for HPS and SEARCH where data was available for direct calculation. Where results for non-fatal events were not available the results for non-fatal coronary events (MRC) or all MI (HYVET, REVEAL) were used in place of non-fatal MI, and all stroke (REVEAL) in place of non-fatal stroke. [b] + implies active treatment better. [c] Report presented results for five cognitive domains; two showed a significant effect.[d] p-values for fatal and non-fatal combined. [e] Results are shown for the aspirin versus placebo arm of the factorial stage. [f] Cognitive population limited to those aged over 65. [g] Significant decline in verbal fluency score (p=0.02, active arm better).

© 2019 Offer A et al. *JAMA Network Open.*

eTable 2. Baseline Factors Considered in Each Study

Baseline factor	How coded	HPS	SEARCH	HPS2-THRIVE	%missing	Comment
Age at entry (single year categories)	G	yes	yes	yes	none	F
Sex	D	yes	yes	yes	none	AF
Height	C	yes	yes	yes	0.04%	
Quintiles of height by sex	G	yes	yes	yes	0.04%	
Townsend deprivation index	C	yes	yes		none	
Quintiles of Townsend deprivation index	G	yes	yes		none	
Weight	C	yes	yes	yes	0.04%	
Quintiles of weight by sex	G	yes	yes	yes	0.04%	
Body mass index (BMI)	C	yes	yes	yes	0.07%	
Grouped BMI (<20,20-,22.5-,25-,27.5, ≥30 kg/m^2)	G	yes	yes	yes	0.07%	
Systolic blood pressure (SBP)	C		yes	yes	0.02%	
Grouped SBP (<120,120-,140-,160-,≥180 mmHg)	G	yes	yes	yes	0.02%	
Diastolic blood pressure (DBP)	C	yes	yes	yes	0.03%	
Grouped DBP (<70,70-,80-,90-,≥100 mmHg)	G	yes	yes	yes	0.03%	
Pulse pressure (PP)	C	yes	yes	yes	0.03%	
Grouped PP (<40,40-,50-,60-,≥70 mmHg)	G	yes	yes	yes	0.03%	
Current smoker	D	yes	yes	yes	none	
Smoking status (current, ex-, never smoker)	G	yes	yes	yes	none	
Drinks alcohol now	D	yes	yes	yes	none	
Alcohol units/week	C	yes	yes	yes	none	
Grouped alcohol units/week (0,<20, ≥20)	G	yes	yes	yes	none	
Prior disease at entry into trial						
Coronary heart disease (MI, other, none)	G	yes	yes	yes	none	AF
Peripheral vascular disease	D	yes	yes	yes	none	AF
Cerebrovascular disease	D	yes	yes	yes	none	AF
Diabetes at entry	D	yes	yes	yes	none	AF
Hypertension treated with drugs	D	yes	yes	yes	none	AF
Heart failure indicator in HPS (N-BNP group)	G	yes			0.58%	AF
Heart failure indicator in HPS2-THRIVE (severity class)	G			yes	none	AF
Hospitalisation for angina	D	yes	yes	yes	none	
Blood measurements at entry into trial						
Apolipoprotein A_1	C	yes	yes	yes	0.06%	
Apolipoprotein B	C	yes	yes	yes	0.07%	
LDL cholesterol	C	yes	yes	yes	0.06%	
HDL cholesterol	C	yes	yes	yes	0.06%	
Total cholesterol	C	yes	yes	yes	0.05%	
Triglycerides	C	yes	yes	yes	0.05%	
Creatinine	C	yes	yes	yes	0.06%	
log(Creatinine)	C	yes	yes	yes	0.06%	
Albumin	C	yes	yes		0.01%	
C-reactive protein	C	yes			10.10%	
log(C-reactive protein)	C	yes			10.10%	
Vitamin B_{12}	C		yes		3.07%	
Estimated glomular filtration rate	C	yes		yes	0.06%	
Folate	C		yes		3.09%	
Homocysteine at randomisation	C		yes		1.26%	
Mean corpuscular volume	C		yes		0.87%	
log(albumin/creatinine ratio)	C			yes	6.47%	
Glycated haemoglobin (HbA1c)	C			yes	3.04%	

© 2019 Offer A et al. *JAMA Network Open.*

eTable 2 (continued)

Baseline factor	How coded	HPS	SEARCH	HPS2-THRIVE	%missing	Comment
Medication at baseline						
ACE inhibitors/ angiotensin receptor blocker	D	yes	yes	yes	none	
Aldosterone antagonist	D	yes	yes	yes	none	
Alpha blocker	D	yes	yes	yes	none	
Aspirin	D	yes	yes	yes	none	
Beta blocker	D	yes	yes	yes	none	
Bronchial dilator	D	yes	yes	yes	none	
Calcium channel blocker	D	yes	yes	yes	none	
Diuretic	D	yes	yes	yes	none	
Insulin	D	yes	yes	yes	none	
Nitrate	D	yes	yes	yes	none	
NSAID / COXIB	D	yes	yes	yes	none	
Warfarin	D	yes	yes	yes	none	
Other anti-platelet medication	D	yes	yes	yes	none	
Traditional chinese medicine	D			yes	none	
Oral hypoglycaemics	D	yes	yes	yes	none	

MI=myocardial infarction, N-BNP=N-terminal Pro-B-type natriuretic peptide, ACE=angiotensin converting enzyme, NSAID/COXIB=Non-steroidal anti-inflammatory drugs/COX-2 inhibitors, D=dichotomous variable, G=grouped (categorical) variable, C=continuous variable, A=included in adjustment of relationship of scores to age, F=forced into stepwise regression.

© 2019 Offer A et al. *JAMA Network Open.*

eTable 3. Baseline Characteristics and Incident Nonfatal In-Trial Events in the 45 029 Participants With Cognitive Function Assessed at the Final Follow-up Visit and in 6944 Participants Surviving to the End of the Trial but With no Cognitive Function Assessment

Characteristic	With cognitive assessment	Without cognitive assessment	All
Participants surviving to the end of the trial	45029	6944	51973
Age (years)			
Age at entry	63.6 (7.96)	64.8 (8.87)	63.7 (8.10)
Grouped age at entry			
< 60	14449 (32%)	2007 (29%)	16456 (32%)
60-70	19532 (43%)	2529 (36%)	22061 (42%)
≥70	11048 (25%)	2408 (35%)	13456 (26%)
Age at end of trial	67.9 (8.03)	70.0 (8.21)	68.1 (8.07)
Baseline characteristics			
Female	8687 (19%)	1837 (26%)	10524 (20%)
Townsend deprivation index[a]	-0.65 (3.12)	0.20 (3.36)	-0.56 (3.16)
Body mass index (kg/m^2)	27.8 (4.31)	27.6 (5.00)	27.8 (4.41)
Systolic blood pressure (mm Hg)	142 (21.8)	144 (23.0)	142 (21.9)
Diastolic blood pressure (mm Hg)	81 (11.5)	80 (12.6)	80.7 (11.6)
Current smoker	6587 (15%)	1290 (19%)	7877 (15%)
Current alcohol drinker	24214 (54%)	2715 (39%)	26929 (52%)
Prior disease at entry			
Myocardial infarction	29530 (66%)	4060 (58%)	33590 (64%)
Other CHD and no myocardial infarction	10991 (24%)	1389 (20%)	12380 (24%)
Peripheral vascular disease	7531 (17%)	1120 (16%)	8651 (17%)
Cerebrovascular disease	8523 (19%)	2294 (33%)	10817 (21%)
Diabetes at entry	10991 (24%)	2048 (29%)	13039 (25%)
Blood measurements at entry (on statin[b])			
LDL cholesterol[b] (mg/dL)	74.5 (24.3)	73.0 (26.3)	74.5 (24.3)
Non-fatal in-trial events			
Disabling Stroke	286 (0.6%)	232 (3.3%)	518 (1.0%)
Mild Stroke (and no disabling stroke)	911 (2.0%)	353 (5.1%)	1264 (2.4%)
Strokes per person[c]	1.14 (0.45)	1.22 (0.56)	1.16 (0.49)
Transient ischaemic attack	872 (1.9%)	165 (2.4%)	1037 (2.0%)
Myocardial infarction	1820 (4.0%)	355 (5.1%)	2175 (4.2%)
Coronary revascularisation	2955 (6.6%)	457 (6.6%)	3412 (6.6%)
Non-coronary revascularisation	1286 (2.9%)	228 (3.3%)	1514 (2.9%)
Heart failure	959 (2.1%)	293 (4.2%)	1252 (2.4%)
Onset of diabetes[d]	2585 (7.6%)	324 (6.6%)	2909 (7.5%)
Gastrointestinal bleed	436 (1.0%)	65 (0.9%)	501 (1.0%)
Other non-vascular events[e]	22339 (49.6%)	3597 (51.8%)	25936 (49.9%)

Data are mean (SD) or number (%) of patients. CHD=coronary heart disease. SI conversion factors: To convert LDL cholesterol to mmol/L, multiply values by 0.0259.

[a] Only available in SEARCH and HPS. [b] At randomisation in HPS (on 40 mg simvastatin) and SEARCH (on 20 mg simvastatin) and at the baseline visit in HPS2-THRIVE (on 40 mg simvastatin +/- ezetimibe). [c] Mean (SD) number of events per person in participants who had at least one event. [d] Percentage is among those at risk of the event. [e] Excluding diabetic complications and neurological and psychiatric events.

© 2019 Offer A et al. *JAMA Network Open.*

eTable 4. Independent Baseline Risk Factors Associated With Cognitive Function in Each Trial From Stepwise Regression

	HPS				SEARCH			
**Baseline factor**	**Effect (SE)**	**Type I p value**	**% total SS**	**Step[a]**	**Effect (SE)**	**Type I p value**	**% total SS**	**Step[a]**
Factors forced in:								
Age at entry, per 10 years[b]		**$6x10^{-192}$**	**5.80%**	0		**$5x10^{-177}$**	**9.96%**	0
Female (vs male)	**0.21 (0.02)**	**$9x10^{-20}$**	**0.45%**	0	**0.22 (0.03)**	**$9x10^{-11}$**	**0.40%**	0
Prior disease at entry								
CHD: myocardial infarction	0.04 (0.02)	0.16	0.02%	0				
CHD: other	0.01 (0.02)							
CHD: none	0.00							
Peripheral vascular disease	**-0.07 (0.02)**	**$5x10^{-6}$**	**0.11%**	0	0.01 (0.07)	0.9	0.00%	0
Cerebrovascular disease	**-0.22 (0.02)**	**$7x10^{-24}$**	**0.55%**	0	**-0.15 (0.04)**	**$5x10^{-4}$**	**0.11%**	0
Diabetes	**-0.09 (0.02)**	**$1x10^{-5}$**	**0.10%**	0	**-0.17 (0.03)**	**$1x10^{-6}$**	**0.23%**	0
Treated hypertension	-0.01 (0.02)	0.4	0.00%	0	-0.02 (0.02)	0.33	0.01%	0
Heart failure indicators:								
N-BNP > 5000 pg/mL	-0.09 (0.03)	0.005	0.08%	0				
N-BNP 2000-5000 pg/mL	-0.07 (0.02)							
N-BNP 1000-2000 pg/mL	-0.02 (0.02)							
N-BNP 400-1000 pg/mL	-0.02 (0.02)							
N-BNP <400 pg/mL	0.00							
Severity class 3/4								
Severity class 2								
Severity class 1								
No heart failure								
Factors selected:								
Townsend deprivation index	**-0.05 (0.00)**	**$7x10^{-93}$**	**2.27%**	1	**-0.03 (0.00)**	**$1x10^{-17}$**	**0.70%**	1
Height, per 10 cm	**0.13 (0.01)**	**$3x10^{-30}$**	**0.70%**	3	**0.12 (0.02)**	**$3x10^{-16}$**	**0.64%**	3
Current smoker (vs other)								
Smoking: Current								
Smoking: Ex-smoker								
Smoking: Never								
Current alcohol drinker								
Alcohol units / week: > 20	**0.27 (0.03)**	**$2x10^{-32}$**	**0.79%**	2	**0.25 (0.03)**	**$4x10^{-18}$**	**0.77%**	2
Alcohol units / week: 1 - 20	**0.15 (0.02)**				**0.16 (0.02)**			
Alcohol units / week: none	**0.00**				**0.00**			
Blood measurements								
LDL-cholesterol, per 20 mg/dL	**-0.06 (0.01)**	**$5x10^{-7}$**	**0.14%**	4				
Homocysteine, per 0.15 mg/L					**-0.01 (0.00)**	**$6x10^{-5}$**	**0.15%**	4
HbA1c, 1% total hemoglobin								
Albumin, per 0.25 g/dL	**0.01 (0.00)**	**$5x10^{-4}$**	**0.06%**	5				
Medication								
ACE inhibitors / ARB								
Aspirin	**0.06 (0.02)**	**$8x10^{-4}$**	**0.06%**	6				
Nitrate								

SS=sum of squares, DBP=diastolic blood pressure, CHD=coronary heart disease, N-BNP=N-terminal Pro-B-type natriuretic peptide, HbA1c=glycated haemoglobin, ACE=angiotensin converting enzyme, ARB=angiotensin receptor blocker. SI conversion factors: To convert LDL cholesterol to mmol/L, multiply values by 0.0259, to convert homocysteine to μmol/L multiply by 7.40

[a] Step=0 indicates the factor was forced into the model. [b]The stepwise model included age in single years as a categorical variable. The effect and SE for age in each study were obtained by running a further regression on the retained variables but with age as a continuous variable.

© 2019 Offer A et al. *JAMA Network Open.*

eTable 4 (continued)

Baseline factor	HPS2-THRIVE (Europe) Effect (SE)	Type I p value	% total SS	Step *	HPS2-THRIVE (China) Effect (SE)	Type I p value	% total SS	Step *
Factors forced in:								
Age at entry, per 10 years[b]		**$1x10^{-165}$**	**6.50 %**	0		**$2x10^{-78}$**	**5.16 %**	0
Female (vs male)	**0.39 (0.03)**	**$3x10^{-37}$**	**1.16 %**	0	**-0.17 (0.04)**	**$8x10^{-6}$**	**0.22 %**	0
Prior disease at entry								
CHD: myocardial infarction	-0.04 (0.03)	0.46	0.01 %	0	**-0.28 (0.04)**	**$5x10^{-14}$**	**0.67 %**	0
CHD: other	-0.01 (0.03)				**0.02 (0.04)**			
CHD: none	0.00				**0.00**			
Peripheral vascular disease	-0.04 (0.02)	0.08	0.02 %	0	0.04 (0.05)	0.47	0.01 %	0
Cerebrovascular disease	**-0.16 (0.03)**	**$3x10^{-10}$**	**0.29 %**	0	**-0.23 (0.03)**	**$8x10^{-16}$**	**0.71 %**	0
Diabetes	-0.03 (0.03)	0.36	0.01 %	0	0.07 (0.03)	0.008	0.08 %	0
Treated hypertension	-0.03 (0.02)	0.08	0.02 %	0	-0.02 (0.02)	0.47	0.01 %	0
Heart failure indicators:								
N-BNP > 5000 pg/mL								
N-BNP 2000-5000 pg/mL								
N-BNP 1000-2000 pg/mL								
N-BNP 400-1000 pg/mL								
N-BNP <400 pg/mL								
Severity class 3/4	-0.53 (0.21)	0.06	0.05 %	0	-0.10 (0.23)	0.06	0.08 %	0
Severity class 2	-0.06 (0.06)				0.07 (0.08)			
Severity class 1	0.02 (0.04)				-0.20 (0.08)			
No heart failure	0.00				0.00			
Factors selected:								
Townsend deprivation index								
Height, per 10 cm	**0.14 (0.01)**	**$2x10^{-28}$**	**0.87 %**	1	**0.16 (0.02)**	**$2x10^{-16}$**	**0.74 %**	1
Current smoker (vs other)	**-0.09 (0.03)**	**$6x10^{-4}$**	**0.08 %**	7				
Smoking: Current					**-0.12 (0.03)**	**$4x10^{-5}$**	**0.22 %**	4
Smoking: Ex-smoker					**-0.11 (0.03)**			
Smoking: Never					**0.00**			
Current alcohol drinker	**0.16 (0.02)**	**$1x10^{-17}$**	**0.52 %**	2				
Alcohol units / week: > 20								
Alcohol units / week: 1 - 20								
Alcohol units / week: none								
Blood measurements								
LDL-cholesterol, per 20 mg/dL								
Homocysteine, per 0.15 mg/L								
HbA1c, per 1% total hemoglobin	**-0.06 (0.01)**	**$7x10^{-7}$**	**0.17 %**	5	**-0.05 (0.01)**	**$2x10^{-5}$**	**0.20 %**	2
Albumin, per 0.25 g/dL								
Medication								

© 2019 Offer A et al. *JAMA Network Open.*

ACE inhibitors / ARB	**-0.09 (0.02)**	**$1x10^{-5}$**	**0.14 %**	6				
Aspirin	**0.14 (0.03)**	**$7x10^{-8}$**	**0.21 %**	4	**0.13 (0.03)**	**$10x10^{-5}$**	**0.16 %**	3
Nitrate	**-0.14 (0.02)**	**$9x10^{-11}$**	**0.30 %**	3	**0.08 (0.02)**	**$7x10^{-4}$**	**0.12 %**	5

SS=sum of squares, DBP=diastolic blood pressure, CHD=coronary heart disease, N-BNP=N-terminal Pro-B-type natriuretic peptide, HbA1c=glycated haemoglobin, ACE=angiotensin converting enzyme, ARB=angiotensin receptor blocker. SI conversion factors: To convert LDL cholesterol to mmol/L, multiply values by 0.0259, to convert homocysteine to μmol/L multiply by 7.40.

[a] Step=0 indicates the factor was forced into the model. [b] The stepwise model included age in single years as a categorical variable. The effect and SE for age in each study were obtained by running a further regression on the retained variables but with age as a continuous variable.

© 2019 Offer A et al. *JAMA Network Open.*

eTable 5. Cognitive Function *z* Score Differences and Cognitive Aging by Randomization to Simvastatin vs Placebo in the Heart Protection Study, Showing the Importance of Adjustment

	All randomized		Surviving to final follow-up	With cognitive assessment					
	N	Mean age at entry	N	N	Mean age at cognitive assessment	Response rate[a] (p value)	Mean (SE) cognitive function z-score	Simvastatin minus placebo: decrease in cognitive aging (95% CI)	p value for difference in cognitive function
Active arm	10269	63.98	8941	8088	68·22	90.46%	0·0024		
Placebo arm	10267	63.95	8760	7838	68·10	89.49%	-0·0025		
Difference using various adjustments									
None	-2	0.03	182	250	0.12	0.98% (0.03)	0·005 (0·016)	0.12 (-0.65, 0.90)	0.76
Age at assessment						0.98% (0.09)	0.009 (0.015)	0.23 (-0.51, 0.98)	0.53
Age + duration in trial and baseline predictors[b]						1.02% (0.08)	0.014 (0.015)	0.35 -0.37, 1.06)	0.35

[a] Percentage of survivors with cognitive assessment. [b] Baseline predictors of cognitive function and major vascular event risk. The excess probability of surviving in the simvastatin arm compared to the placebo arm was greater at older ages, resulting in a 0.12 years higher mean age at cognitive assessment in the simvastatin arm. Unadjusted analyses (previously reported[21]) fail to allow for this, leading to an approximately similar underestimation of the decrease in cognitive aging with simvastatin.

© 2019 Offer A et al. *JAMA Network Open.*

eTable 6. Sensitivity Analysis to Show the Effect of Assuming Different Rates of Declines in Cognitive Function With Age

	Reduction in years of cognitive aging with allocation to statin by assumed % SD decline in cognitive function by year of age		
	**3%**	**4%**	**5%**
**Per Event**			
Stroke: disabling	12.4	9.3	7.5
Stroke: mild or unknown disability	8.5	6.3	5.1
Transient ischaemic attack	3.2	2.4	1.9
Myocardial infarction	2.1	1.5	1.2
Heart failure	2.6	2.0	1.6
New onset diabetes	1.8	1.3	1.1
**Reduction in years of cognitive aging / person**			
All survivors: predicted effect from events avoided	0.20	0.15	0.12
Survivors with cognitive assessment: predicted effect from events avoided	0.19	0.14	0.11
**Observed difference**	0.46 (95%CI: -0.49, 1.41)	0.35 (95%CI: -0.37, 1.06)	0.28 (95%CI: -0.30, 0.85)

© 2019 Offer A et al. *JAMA Network Open.*

eFigure 1. Study Profiles of Cohorts Included in the First Stage of the Analysis

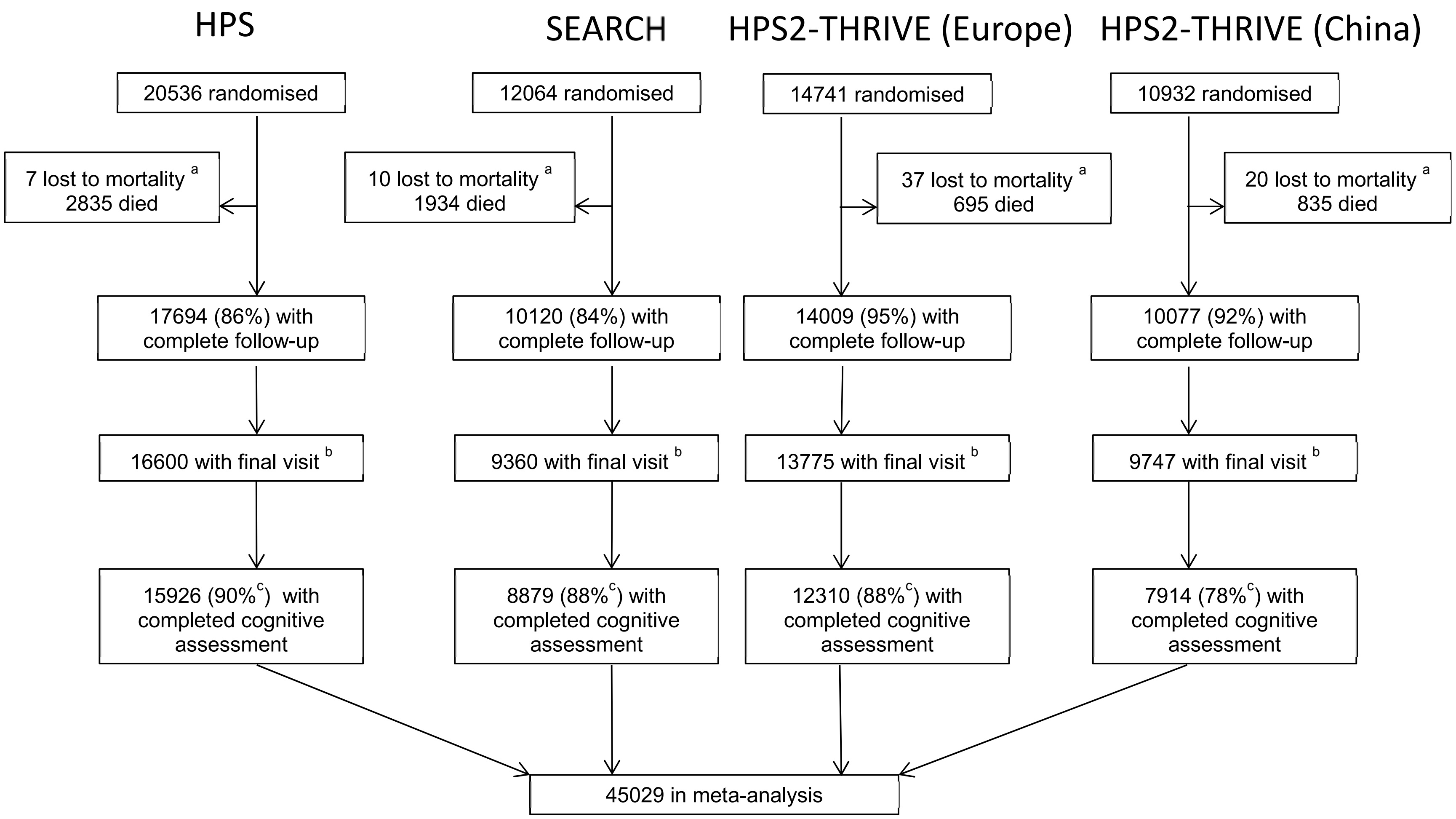

[a] Numbers lost to follow-up relate to those without information to the end of the scheduled treatment period for mortality alone. [b] In person or by telephone.
[c] Percentage of those not known to have died that completed the modified telephone interview for cognitive status and verbal fluency test.

© 2019 Offer A et al. *JAMA Network Open.*

eFigure 2. Study Profile by Randomization Arm of HPS, the Only Study Eligible for the Second Stage of the Analysis

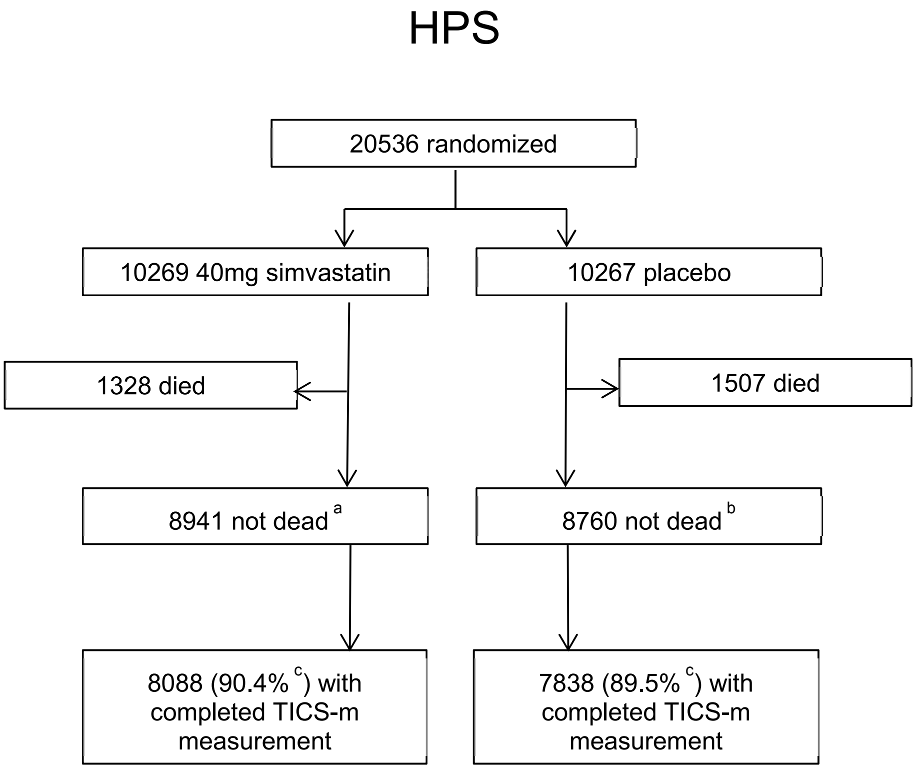

[a] Including 3 participants lost to mortality. [b] Including 4 participants lost to mortality. [c] Percentage of those not known to have died that completed the modified telephone interview for cognitive status and verbal fluency test.

© 2019 Offer A et al. *JAMA Network Open.*

eFigure 3. Associations of the Components of the Global Cognitive Function Score With Age

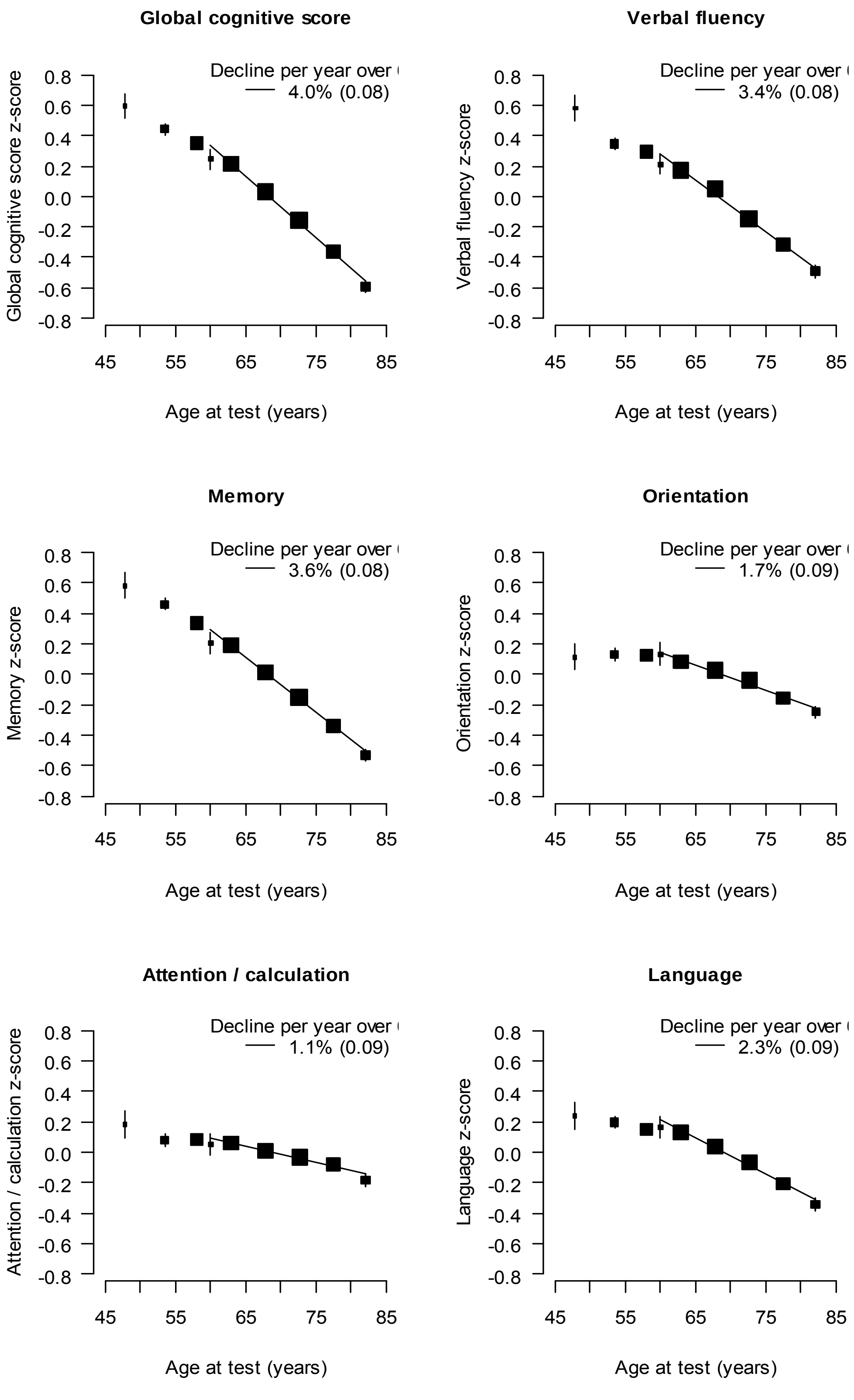

Adjusted for prior disease, sex and study cohort

© 2019 Offer A et al. *JAMA Network Open.*

eFigure 4. Cognitive Aging Associated With the Incidence of Nonfatal Cerebrovascular Events by Time From Event to Assessment and by Age at Assessment

Non-fatal incident event	N	Cognitive function z-score difference (95% CI)	Equivalent years of cognitive ageing (95% CI)	p value
**By event timing**				
**Stroke**				
Over 2 years before assessment	686	-0.29 (-0.37, -0.20)	7.1 (5.1, 9.1)	*<0.001*
Within 2 years of assessment	543	-0.27 (-0.36, -0.17)	6.7 (4.3, 9.0)	*<0.001*
**Transient ischaemic attack**				
Over 2 years before assessment	508	-0.08 (-0.18, 0.01)	2.1 (-0.3, 4.5)	*0.09*
Within 2 years of assessment	401	-0.10 (-0.19, 0.00)	2.4 (-0.1, 4.8)	*0.06*
**By age at assessment**				
**Stroke**				
<60	145	-0.26 (-0.44, -0.08)	6.4 (1.9, 10.9)	*0.005*
60-70	443	-0.32 (-0.42, -0.22)	7.9 (5.4, 10.4)	*<0.001*
≥ 70	609	-0.27 (-0.36, -0.18)	6.7 (4.5, 8.9)	*<0.001*
**Transient ischaemic attack**				
<60	99	-0.09 (-0.29, 0.11)	2.3 (-2.8, 7.3)	*0.38*
60-70	287	-0.10 (-0.22, 0.02)	2.6 (-0.5, 5.6)	*0.10*
≥ 70	496	-0.10 (-0.20, -0.01)	2.6 (0.2, 5.0)	*0.03*

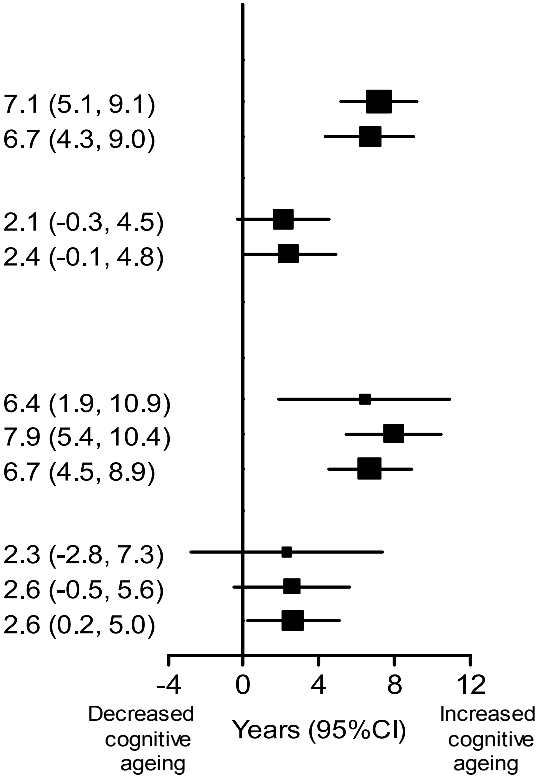

Analysis details as for figure 2

© 2019 Offer A et al. *JAMA Network Open.*

eReferences

1. Power MC, Weuve J, Richey Sharrett A, Blacker D, Gottesman RF. Statins, cognition, and dementia—systematic review and methodological commentary. *Nat. Rev. Neurol.* 2015; **11**: 220–229.

2. McGuinness B, Craig D, Bullock R, Passmore P. Statins for the prevention of dementia. *Cochrane Database of Systematic Reviews* 2016; Issue **1**: Art. No.: CD003160.

3. Zanchetti A1, Liu L, Mancia G, Parati G, Grassi G, Stramba-Badiale M, Silani V, Bilo G, Corrao G, Zambon A, Scotti L, Zhang X, Wang H, Zhang Y, Zhang X, Guan TR, Berge E, Redon J, Narkiewicz K, Dominiczak A, Nilsson P, Viigimaa M, Laurent S, Agabiti-Rosei E, Wu Z, Zhu D, Rodicio JL, Ruilope LM, Martell-Claros N, Pinto F, Schmieder RE, Burnier M, Banach M, Cifkova R, Farsang C, Konradi A, Lazareva I, Sirenko Y, Dorobantu M, Postadzhiyan A, Accetto R, Jelakovic B, Lovic D, Manolis AJ, Stylianou P, Erdine S, Dicker D, Wei G, Xu C, Xie H, Coca A, O'Brien J, Ford G. Blood pressure and LDL-cholesterol targets for prevention of recurrent strokes and cognitive decline in the hypertensive patient: design of the European Society of Hypertension-Chinese Hypertension League Stroke in Hypertension Optimal Treatment randomized trial. *Journal of Hypertension* 2014; **32**: 1741–1750

4. Rouch, L., Cestac, P., Hanon, O. Cool C, Helmer C, Bouhanick B, Chamontin B, Dartigues JF, Vellas B, Andrieu S. Antihypertensive drugs, prevention of cognitive decline and dementia: a systematic review of observational studies, randomized controlled trials and meta-analyses, with discussion of potential mechanisms. *CNS Drugs* 2015; **29**: 113-130.

5. McGuinness B, Todd S, Passmore P, Bullock R. Blood pressure lowering in patients without prior cerebrovascular disease for prevention of cognitive impairment and dementia.*Cochrane Database of Systematic Reviews* 2009; Issue **4**: Art. No.: CD004034

6. PROGRESS Collaborative Group. Randomised trial of a perindopril-based blood-pressure-lowering regimen among 6105 individuals with previous stroke or transient ischaemic attack. *Lancet* 2001; **358**: 1033–1041 .

7. Tzourio C, Anderson C, Chapman N, Woodward M, Neal B, MacMahon S, Chalmers J, PROGRESS Collaborative Group. Effects of blood pressure lowering with perindopril and indapamide therapy on dementia and cognitive decline in patients with cerebrovascular disease. *Arch Intern Med* 2003; **163**: 1069–1075

© 2019 Offer A et al. *JAMA Network Open.*

8. Applegate WB, Pressel S, Wittes J, Luhr J, Shekelle RB, Camel GH, Greenlick MR, Hadley E, Moye L, Perry HM Jr, Scfron E, Wegener V. Impact of the treatment of isolated systolic hypertension on behavioural variables. Results from the Systolic Hypertension in the Elderly Program. *Arch Intern Med* 1994; **154**: 2154–2160**.**

9. SHEP Cooperative Research Group Prevention of Stroke by Antihypertensive Drug Treatment in Older Persons With Isolated Systolic Hypertension Final Results of the Systolic Hypertension in the Elderly Program (SHEP). *JAMA*. 1991; **265**: 3255-3264.

10. Lithell H, Hansson L, Skoog I, Elmfeldt D, Hofman A, Olofsson B, Trenkwalder P, Zanchetti A; SCOPE Study Group. The Study on Cognition and Prognosis in the Elderly (SCOPE): principal results of a randomised double-blind intervention trial. *J Hypertens* 2003; **21**: 875–886.

11. Skoog I, Lithell H, Hansson L, Elmfeldt D, Hofman A, Olofsson B, Trenkwalder P, Zanchetti A; SCOPE Study Group. Effect of baseline cognitive function and antihypertensive treatment on cognitive and cardiovascular outcomes: Study on Cognition and Prognosis in the Elderly (SCOPE). *Am J Hypertens* 2005; **18:** 1052–1059.

12. B.K. Saxby BK, Harrington F, Wesnes KA, McKeith IG, Ford GA. Candesartan and cognitive decline in older patients with hypertension - A substudy of the SCOPE trial. *Neurology*. 2008; **70**: 1858-1866

13. Staessen JA, Fagard R, Thijs L, Celis H, Arabidze GG, Birkenhäger WH, Bulpitt CJ, de Leeuw PW, Dollery CT, Fletcher AE, Forette F, Leonetti G, Nachev C, O'Brien ET, Rosenfeld J, Rodicio JL, Tuomilehto J, Zanchetti A. Randomised double-blind comparison of placebo and active treatment for older patients with isolated systolic hypertension. *Lancet* 1997; **350:** 757–64 (correction: *Lancet* 1997; **350:** 1636).

14. Forette F, Seux ML, Staessen JA, Thijs L, Birkenhäger WH, Babarskiene MR, Babeanu S, Bossini A, Gil-Extremera B, Girerd X, Laks T, Lilov E, Moisseyev V, Tuomilehto J, Vanhanen H, Webster J, Yodfat Y, Fagard R. Prevention of dementia in randomised double-blind placebo-controlled Systolic Hypertension in Europe (Syst-Eur) trial.. *Lancet* 1998; **352**: 1347–1351

15. MRC Working Party. Medical Research Council trial of treatment of hypertension in older adults: principal results. *BMJ* 1992; **304**: 405-412.

© 2019 Offer A et al. *JAMA Network Open.*

16. Prince MJ, Bird AS, Blizard RA, Mann AH. Is the cognitive function of older patients affected by antihypertensive treatment? Results from 54 months of the Medical Research Council's treatment trial of hypertension in older adults. *BMJ* 1996; **312**: 801–805.

17. The ACCORD Study Group Effects of Intensive Blood-Pressure Control in Type 2 Diabetes Mellitus *N Engl J Med* 2010; **362**: 1575-1585.

18. Williamson JD, Launer LJ, Bryan RN, Coker LH, Lazar RM, Gerstein HC, Murray AM, Sullivan MD, Horowitz KR, Ding J, Marcovina S, Lovato L, Lovato J, Margolis KL, Davatzikos C, Barzilay J, Ginsberg HN, Linz PE, Miller ME; Action to Control Cardiovascular Risk in Diabetes Memory in Diabetes Investigators. Cognitive Function and Brain Structure in Persons With Type 2 Diabetes Mellitus After Intensive Lowering of Blood Pressure and Lipid Levels - A Randomised Clinical Trial *JAMA Intern Med.* 2014; **174**: 324-333.

19. Beckett N, Peters R, Fletcher A, Jan A, Staessen JA, Liu L, Dumitrascu D, Stoyanovsky V, Antikainen RL, Nikitin Y, Anderson C, Belhani A, Forette F, Rajkumar C, Thijs L, Banya W, Bulpitt CJ; HYVET Study Group. Treatment of hypertension in patients 80 years of age or older. *N Engl J Med* 2008; **358:** 1887–1898.

20. Peters R, Beckett N, Forette F, Tuomilehto J, Clarke R, Ritchie C, Waldman A, Walton I, Poulter R, Ma S, Comsa M, Burch L, Fletcher A, Bulpitt C; HYVET investigators. Incident dementia and blood pressure lowering in the Hypertension in the Very Elderly Trial cognitive function assessment (HYVETCOG): a double-blind, placebo controlled trial. *Lancet Neurol* 2008; **7:** 683–689.

21. Heart Protection Study Collaborative Group. MRC/BHF Heart Protection Study of cholesterol lowering with simvastatin in 20,536 high-risk individuals: a randomised placebo-controlled trial. *Lancet.* 2002; **360**: 7-22.

22. SEARCH Collaborative Group. Intensive lowering of LDL cholesterol with 80 mg versus 20 mg simvastatin daily in 12,064 survivors of myocardial infarction: a double-blind randomised trial. Lancet. 2010; **376**: 1658-1669.

23. Sabatine MS, Giugliano RP, Keech AC, Honarpour N, Wiviott SD, Murphy SA, Kuder JF, Wang H, Liu T, Wasserman SM, Sever PS, Pedersen TR; FOURIER Steering Committee and Investigators. Evolocumab and Clinical Outcomes in Patients with Cardiovascular Disease *N Engl J Med* 2017; **376**: 1713-22.

© 2019 Offer A et al. *JAMA Network Open.*

24. Guigliano RP, Mach F, Zavitz K, Kurtz C, Im K, Kanevsky E, Schneider J, Wang H, Keech A, Pedersen TR, Sabatine MS, Sever PS, Robinson JG, Honarpour N, Wasserman SM, Ott BR; EBBINGHAUS Investigators.. Cognitive Function in a Randomised Trial of Evolocumab. *N Engl J Med* 2017; **377**: 633-643.

25. Shepherd J, Blauw GJ, Murphy MB, Bollen ELEM, Buckley BM, Cobbe SM, Ford I, Gaw A, Hyland M, Jukema JW, Kamper AM, Macfarlane PW, Meinders AE, Norrie J, Packard CJ, Perry IJ, Stott DJ, Sweeney BJ, Twomey C, Westendorp RG; PROSPER study group. PROspective Study of Pravastatin in the Elderly at Risk. Pravastatin in elderly individuals at risk of vascular disease (PROSPER): a randomised controlled trial. *Lancet* 2002; **360**: 1623–1630.

26. The HPS3 / TIMI55 – REVEAL Collaborative Group. Effects of Anacetrapib in Patients with Atherosclerotic Vascular Disease. *N Engl J Med* 2017; **00**: 000-000 DOI:10.1056/NEJMoa1706444

27. The Medical Research Council's General Practice Research Framework . Thrombosis prevention trial: randomised trial of low-intensity oral anticoagulation with warfarin and low-dose aspirin in the primary prevention of ischaemic heart disease in men at increased risk. *Lancet* 1998; **351**: 233–241 .

28. Richards M, Meade TW, Peart S, Brennan PJ, Mannet AH. Is there any evidence for a protective effect of antithrombotic medication on cognitive function in men at risk of cardiovascular disease? Some preliminary findings *Journal of Neurology, Neurosurgery, and Psychiatry* 1997; **62:** 269-272.

29. Ridker PM, Cook NR, Lee I-M, Gordon D, Gaziano JM, Manson JE, Hennekens CH, Buring JE. A Randomised Trial of Low-Dose Aspirin in the Primary Prevention of Cardiovascular Disease in Women *N Engl J Med* 2005; **352**: 1293-304.

30. Kang JH, Cook N, Manson J, Buring JE, and Grodstein F. Low dose aspirin and cognitive function in the women's health study cognitive cohort *BMJ* 2007*;* **334***:* 987-990.

31. Iadecola C, Yaffe K, Biller J, Bratzke LC, Faraci FM, Gorelick PB, Gulati M, Kamel H, Knopman DS, Launer LJ, Saczynski JS, Seshadri S, Zeki Al Hazzouri A. Impact of Hypertension on Cognitive Function - A Scientific Statement From the American Heart Association. *Hypertension*. 2016; **68**:000-000. DOI: 10.1161/HYP.0000000000000053.

© 2019 Offer A et al. *JAMA Network Open.*

32. Heart Protection Study Protocol. Available from: http://www.hpsinfo.org [Accessed 20th October 2017]
33. SEARCH Protocol. Available from: https://www.ctsu.ox.ac.uk/research/search [Accessed 20th October 2017]
34. HPS2-THRIVE Collaborative Group. Effects of extended-release niacin with laropiprant in high-risk patients. N Engl J Med 2014; **371**: 203-212.
35. Heart Protection Study Collaborative Group. MRC/BHF Heart Protection Study of cholesterol lowering and of antioxidant vitamin supplementation in a wide range of patients at increased risk of heart disease death: early safety and efficacy experience. *Eur Heart J* 1999; **20**: 725-741.
36. SEARCH Collaborative Group. Study of the effectiveness of additional reductions in cholesterol and homocysteine (SEARCH): characteristics of a randomised trial among 12064 myocardial infarction survivors. Am Heart J 2007; **154**: 815-823, 23 e1-6.
37. HPS2-THRIVE Collaborative Group. HPS2-THRIVE randomised placebo-controlled trial in 25 673 high-risk patients of ER niacin/laropiprant: trial design, pre-specified muscle and liver outcomes, and reasons for stopping study treatment. Eur Heart J 2013; **34**: 1279-1291.
38. Lyall DM, Cullen B, Allerhand M, Smith DJ, Mackay D, Evans J, Anderson J, Fawns-Ritchie C, McIntosh AM, Deary IJ, Pell JP. Cognitive Test Scores in UK Biobank: Data Reduction in 480,416 Participants and Longitudinal Stability in 20,346 Participants. PLoS One 2016; **11**: e0154222 10.1371/journal.pone.0154222.
39. Clarke R, Bennett D, Parish S, Lewington S, Skeaff M, Eussen SJ, Lewerin C, Stott DJ, Armitage J, Hankey GJ, Lonn E, Spence JD, Galan P, de Groot LC, Halsey J, Dangour AD, Collins R, Grodstein F; B-Vitamin Treatment Trialists' Collaboration. Effects of homocysteine lowering with B vitamins on cognitive aging: meta-analysis of 11 trials with cognitive data on 22,000 individuals. American Journal of Clinical Nutrition. 2014; **100**: 657-666.
40. Heart Protection Study Collaborative Group. Cost-effectiveness of simvastatin in people at different levels of vascular disease risk: economic analysis of a randomised trial in 20,536 individuals. Lancet 2005*;* **365**: 1779-1785.
41. Borm GF, Fransen J, Lemmens WAJG. A simple size formula for analysis of covariance in randomised clinical trials. Journal of Clinical Epidemiology 2007; **60**: 1234-1238.
42. Giugliano RP, Sabatine MS, Ott BR. Cognitive Function in a Randomized Trial of Evolocumab. N Engl J Med. 2017; **377**(20): 1997.

© 2019 Offer A et al. *JAMA Network Open.*
